# A Retrospective Analysis of the Changes in Prediabetes-Associated Markers of Thyroid Function in Patients from Durban, South Africa

**DOI:** 10.3390/ijms26052170

**Published:** 2025-02-28

**Authors:** Hasnaa Satar Aswani, Wendy Mdluli, Andile Khathi

**Affiliations:** Department of Human Physiology, School of Laboratory Medicine and Medical Sciences, College of Health Sciences, University of KwaZulu-Natal, Durban 3629, South Africa; 219039948@stu.ukzn.ac.za (W.M.); khathia@ukzn.ac.za (A.K.)

**Keywords:** prediabetes, thyroid function, type 2 diabetes melittus, Durban, South Africa

## Abstract

Thyroid dysfunction and type 2 diabetes melittus (T2DM) are two of the most common endocrine disorders, and the emerging condition of prediabetes necessitates additional research to better understand the complex interactions between thyroid hormones, metabolic regulation, and the progression from prediabetes to T2DM. This study sought to investigate changes in selected markers of thyroid function in patients with pre-diabetes. Upon obtaining ethics permission, blood samples were collected from patients in King Edward Hospital in Durban, South Africa. The samples were classified as non-diabetic, pre-diabetic, and type 2 diabetic using the ADA guidelines. The thyroid stimulating hormone (TSH), thyroxine (T4) triiodothyronine (T3), Thyroglobulin (TG), and thyroid peroxidase antibody (TPOAb) concentrations were determined in these samples. The results showed elevated TSH, decreased T3 and T4, decreased thyroglobulin (Tg), and elevated TPOAb in the prediabetic group which became considerably pronounced with the shift to T2DM. The alterations in these markers during prediabetes may indicate an early stage of thyroid dysfunction necessitating further investigation as these alterations become more pronounced during type 2 diabetes mellitus.

## 1. Introduction

The maintenance of glucose homeostasis requires an interplay between glucose production and peripheral glucose utilization [1]. Glucose homeostasis influences thyroid function by modulating insulin signaling and deiodinase activity, which affect thyroid hormone conversion and HPT axis regulation, while thyroid hormones, in turn, regulate glucose metabolism by enhancing insulin sensitivity, glucose uptake, and energy balance [2,3,4,5].

The secretion of thyroid hormones from the thyroid gland is regulated by the hypothalamic–pituitary axis, where stimulation of the thyroid gland by thyroid stimulating hormone (TSH) results in the secretion of both triiodothyronine (T3) and thyroxine (T4) in the circulation [3]. T3 and T4, along with thyroid peroxidase antibody (TPOAb), can, directly and indirectly, regulate insulin secretion either by reducing glucose-induced insulin secretion or by reducing beta-cell responsiveness that results in hypo- and hyperthyroidism, respectively [4,6,7,8].

The relationship between thyroid disorders and T2DM has been a subject of ongoing interest as they are treated concurrently in medical practice today [9,10]. A 7-year prospective study conducted in Korea reported that the risk of T2DM was positively associated with the level of TSH, while negatively associated with the level of T3 and FT4. Other cross-sectional studies in Europe suggested that thyroid hormones were positively associated with the prevalence and incidence of T2DM [11,12,13]. Several studies show that even a modest increase in TSH is associated with sub-clinical hypothyroidism and is correlated with insulin resistance that plays a role in the pathogenesis of T2DM [5,14,15,16]. These contradictory findings from previous studies suggest there are more complex pathophysiologic mechanisms linking the relationship between thyroid hormones and glucose metabolism.

T2DM has been shown to reduce thyroid-stimulating hormone levels and impair the conversion of thyroxine (T4) to triiodothyronine (T3) in the peripheral tissues [9]. Furthermore, variations of thyroid function within the normal range might be associated with the development and progression of T2DM [11,12,17]. Pre-diabetes is a condition of metabolism characterized by slight glycaemic dysregulation and is a risk factor for the development of T2DM and the associated complications [18,19].

Currently, the global population with prediabetes has been estimated to be approximately 318 million, although the number could be much higher as the condition goes undiagnosed due to its asymptomatic nature [20]. About 69.2% of the people with prediabetes live in low- and middle-income countries, such as those found in Africa [20,21]. The rising prevalence of prediabetes could be attributed to rapid urbanization that includes sedentary lifestyles and chronic consumption of high-calorie diets [22].

A recent systematic review and meta-analysis indicated that the overall prevalence of prediabetes was 15.56% in individuals aged between 25 and 45 years in the South African population [23]. The study also showed that females have an 18% greater chance of developing prediabetes when compared to males [23]. Interestingly, studies have also mentioned that thyroid dysfunction rises with age and is more prevalent in females when compared to males [14,24]. Indeed, a retrospective study performed by Sosibo and colleagues indicated an alarmingly high prevalence of prediabetes in individuals aged between 25 and 45 years old in the South African population [25].

However, no study has been performed in South Africa to investigate the association between prediabetes and thyroid dysfunction. While a previous study was conducted to investigate the association, it was conducted in a diet-induced animal model of prediabetes [26]. There is a lack of data on this association in a clinical-based setting; hence, this study sought to investigate the changes in selected markers of thyroid function in prediabetic individuals.

## 2. Results

**Figure 1:**
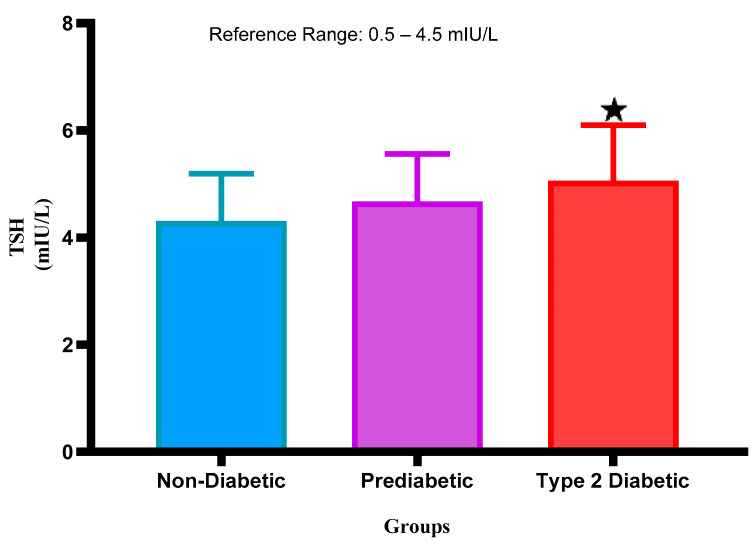
Thyroid stimulating hormone concentration was measured in plasma of ND, PD, and T2DM. Values are presented as mean and standard deviation. * *p* < 0.05 in comparison with the non-diabetic control group.

**Figure 2:**
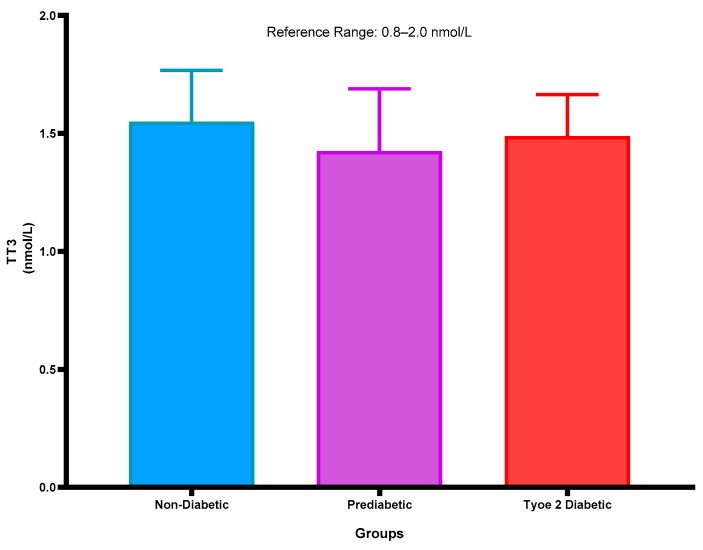
Total triiodothyronine concentration that was measured in plasma of ND, PD, and T2DM. Values are presented as mean and standard deviation.

**Figure 3:**
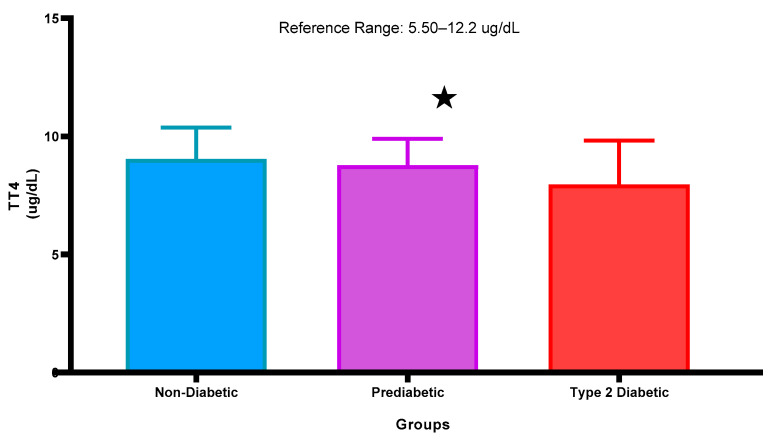
Total thyroxine concentration that was measured in plasma of ND, PD, and T2DM. Values are presented as mean and standard deviation. * *p* < 0.05 in comparison with the non-diabetic control group.

**Figure 4:**
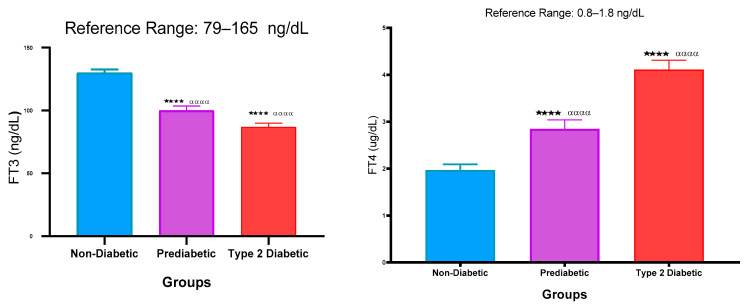
Free triiodothyronine and free thyroxine concentration that was measured in plasma of ND, PD, and T2DM. Values are presented as mean and standard deviation. *p* < 0.001 in comparison with the non-diabetic control group. **** *p* < 0.001 in comparison with the non-prediabetic group. and αααα *p* < 0.001 in comparison with the prediabetic group.

**Figure 5:**
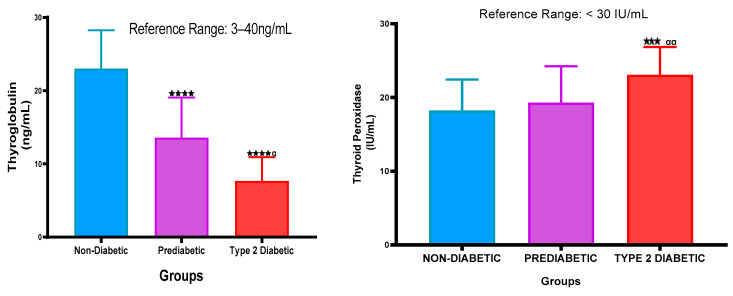
Thyroglobulin and Thyroid Peroxidase Antibody concentration was measured in plasma of ND, PD, and T2DM. Values are presented as mean and standard deviation. *** *p* < 0.003 in comparison with the non-prediabetic control group. **** *p* < 0.001 in comparison with the non-prediabetic group. α *p* < 0.05, αα *p* < 0.01 in comparison with the prediabetic group.

The study examined various thyroid hormone imbalances associated with type 2 diabetes meilttus in individuals with prediabetes and compared to those who were non-pre-diabetic and type 2 diabetic. This was done using ELISA analyses to assess the relevant markers. Particpants characteristics are detailed in Table 1, while Figure 1, Figure 2, Figure 3, Figure 4 and Figure 5 presents the ELISA results for thyroid function (TSH, T3, T4, FT3, FT4, TG and TPOAb). Additionally, spearman correlation analysis in Table 2 and Table 3 show the correlations between thyroid markers and HbA1c and FBG respectively.

Table A1 presents thyroid hormone changes stratified by gender, highlighting differences in hormone concentrations between male and female participants. Due to a small sample size, there was an unequal gender distribution which led to an inconclusion on a potential gender-based variation in thyroid function.

**Table 1:** Characteristics of participants involved in the analysis of thyroid hormonal changes.

	Non-Pre-Diabetic	Prediabetic	Type 2 Diabetic
Age (mean)	37.69 (34.80–40.58)	37.70 (35.34–40.06)	38.48 (36.12–40.85)
Sex			
Male	7	21	16
Female	20	7	12
Fasting Glucose median (IQR)	4.9 mmol/L (4.6–5.5)	6.4 mmol/L (6.0–6.9)	8.5 mmol/L (7.6–9.75)
HbA1c mean (95% CI)	5.24% (5.135–5.35)	6.08% (5.985–6.178)	10.78% (9.506–12.05)

The data are presented as mean (95% CI) or median (IQR) depending on the normality and proportion.

### 2.1. Thyroid Stimulating Hormone (TSH) Concentration

Figure 1 represents the TSH concentration in the plasma of NPD, PD, and T2DM patients. The TSH concentrations were within the normal range of the PD and were slightly elevated in comparison with the NPD. Furthermore, in comparison with the T2DM, it was observed that the TSH concentration was elevated in comparison with the PD group. Interestingly, the TSH concentration increased significantly (*p* < 0.05) in the T2DM group in comparison with the non-diabetic control. See Figure 1.

### 2.2. Total Triiodothyronine (T3) Concentration

Figure 2 represents the T3 concentration in the plasma of NPD, PD, and T2DM patients. The T3 concentration was within the normal range of the PD and was decreased slightly non-significantly in comparison with the NPD. Furthermore, in comparison with T2DM, it was observed that the T3 concentration was slightly elevated in comparison with the PD group. See Figure 2.

### 2.3. Total Thyroxine Concentrations

Figure 3 represents the T4 concentration in the plasma of NPD, PD, and T2DM patients. The T4 concentrations are within the normal range of all groups. The T4 concentration of the PD was decreased slightly non-significantly in comparison with the NPD. Furthermore, in comparison with the T2DM, it was observed that the T4 concentration was slightly decreased in comparison with the PD group. Interestingly, the T4 concentration decreased significantly (*p* < 0.05) in the T2DM group in comparison with the non-diabetic control. See Figure 3.

### 2.4. Free Triiodothyronine (FT3) and Free Thyroxine (FT4) Concentration

Figure 4 represents the FT3 and FT4 concentrations in the plasma of NPD, PD, and T2DM patients. The FT3 concentration of the PD was decreased significantly (*p* < 0.001) in comparison with the NPD. Furthermore, in comparison with the T2DM group, the FT3 concentration also decreased significantly (*p* < 0.001) in comparison with the PD. Additionally, there was a significant (*p* < 0.001) decrease in the T2DM group when compared to the PD group. The FT4 concentration of the PD was significantly elevated (*p* < 0.001) in comparison with the NPD. Additionally, it was observed that there was a significant (*p* < 0.01) increase in the T2DM group in comparison with the PD. The FT4 concentration also increased significantly (*p* < 0.001) in the T2DM group in comparison with the non-diabetic control.

### 2.5. Thyroglobulin (TG) and Thyroid Peroxidase Antibody Concentration

Figure 5 represents the TG and TPOAb concentration in the plasma of NPD, PD, and T2DM patients. The values of TG were within the normal range in all groups. The TG concentration of the PD was decreased significantly (*p* < 0.001) in comparison with the NPD. Furthermore, in comparison with the T2DM group, the TG concentration also decreased significantly (*p* < 0.05) in comparison with the PD. Additionally, there was a significant (*p* < 0.001) decrease in the T2DM group when compared to the PD group. The TPOAb concentration of the PD was elevated in comparison with the NPD. Additionally, it was observed that there was a significant (*p* < 0.05) increase in the T2DM group in comparison with the PD. The TPOAb concentration also increased significantly (*p* < 0.001) in the T2DM group in comparison with the non-diabetic control. See Figure 5.

**Table 2:** Correlation coefficients (r-values) between HbA1c and thyroid function markers.

Group	Biomarker				
	Thyroid Stimulating Hormone	Triiodothyronine	Thyroxine	Thyroglobulin	Thyroid Peroxidase Antibody
PD	−0.09	−0.30	−0.14	0.09	0.05
T2DM	0.27	−0.40	0.17	0.23	−0.24

**Table 3:** Correlation coefficients (r-values) between FPG and thyroid function markers.

Group	Biomarker				
	Thyroid Stimulating Hormone	Triiodothyronine	Thyroxine	Thyroglobulin	Thyroid Peroxidase Antibody
PD	−0.30	0.20	0.12	0.60	−0.10
T2DM	0.25	-0.50	0.47	0.14	−0.20

## 3. Discussion

Thyroid hormones, primarily triiodothyronine (T3) and thyroxine (T4), play a crucial role in regulating metabolic processes influencing carbohydrate metabolism, which is vital for maintaining glucose homeostasis [27,28]. Thyroid dysfunction and T2DM are meticulously correlated endocrine disorders with a significant overlap in prevalence [29]. In individuals with T2DM, the presence of insulin resistance can lead to alterations in thyroid hormone levels, which may exacerbate metabolic dysregulation and contribute to further complications [30,31]. This bidirectional relationship is influenced by common risk factors like obesity, age, and autoimmunity [30]. Notably, complications associated with T2DM can begin to manifest during prediabetes, a condition characterized by moderately- impaired fasting glucose and glucose tolerance as well as elevated concentration of glycated hemoglobin [18,32]. Previous studies using animal models have shown an association between prediabetes and thyroid hormone imbalances, leading to sub-clinical hypothyroidism or hyperthyroidism [26]. The city of Durban in South Africa is characterized by a diverse population that reflects a range of lifestyle factors and genetic predispositions. A retrospective study conducted by Sosibo et al. indicated an alarmingly high prevalence of prediabetes in individuals aged between 25–45 years old in South Africa [25]. Another study conducted in a Durban-based setting also found that patients with prediabetes exhibited significant hormonal imbalances which are linked to increased insulin resistance, however, the changes in thyroid hormones were not investigated [33]. Therefore, using this population, this study sought to assess any changes in thyroid function in patients with prediabetes.

The American Diabetes Association (ADA) has established specific criteria for diagnosing type 2 diabetes mellitus (T2DM) and prediabetes [34]. The ADA defines prediabetes as a state characterized by impaired fasting glucose (IFG), impaired glucose tolerance (IGT), and/or elevated hemoglobin A1c (HbA1c) levels [34]. Specifically, IFG is indicated by fasting plasma glucose levels ranging from 5.6–6.9 mmol/L in prediabetes and 7.0 mmol/L or higher in T2DM, while HbA1c levels between 5.7% and 6.4% for prediabetes and 6.5% or higher for T2DM [34]. Indeed, the results presented in this study fasting glucose and HbA1c values consistent with those defined by ADA for both prediabetes and T2DM. The rapid urbanization observed in this population has been shown to be a contributing factor in the increasing prevalence of both prediabetes and T2DM [35]. This was associated with increased consumption of high-calorie diets combined with increasingly sedentary lifestyles [35]. These conditions have been further shown to dysregulate endocrine dysfunction [33].

The secretion of thyroxine (T4) and triiodothyronine (T3) is controlled by a complex interaction involving other hormones and the functioning of the thyroid gland [5]. The relationship between TSH and thyroid hormones operates through a negative feedback loop where elevated levels of T3 and T4 inhibit TSH secretion from the anterior pituitary gland [36]. However, pathophysiological changes can disrupt this balance [37]. The pathophysiology of TSH changes during T2DM is multifaceted and involves several mechanisms [30,38]. Specifically, studies have shown that elevated glucose concentrations, as seen in T2DM, can downregulate the expression of iodothyronine deiodinase type 2 (DIO2), an enzyme critical for converting T4 to the more active T3 [38]. This reduction in DIO2 activity results in decreased intracellular concentrations of T3, which can further impair glucose metabolism and exacerbate insulin resistance, creating a vicious cycle that affects TSH levels [38]. During T2DM, the presence of insulin resistance can lead to a compensatory increase in TSH as the body attempts to stimulate the thyroid gland to produce more T3 and T4 to counteract the effects of insulin resistance [39]. Indeed, the results presented in this study show a statistically significant increase in TSH concentrations measured in T2DM patients in comparison with non-prediabetic patients. The findings in this study appear to extend this trend to the prediabetic state as we observed slightly elevated TSH concentrations in the prediabetic group in comparison with the non-prediabetic group. Interestingly, this increase in TSH was also seen when we compared the prediabetic group to the T2DM group. The findings of this study corroborate previous studies that have shown that patients with prediabetes may exhibit elevated TSH concentrations, suggesting a potential early disruption in thyroid function that could progress to overt thyroid dysfunction as insulin resistance worsens [15,30,40]. In prediabetes, it has been observed that TSH concentration may increase, potentially due to the interplay between insulin resistance and the hypothalamic–pituitary–thyroid (HPT) axis [26].

Thyroglobulin (Tg) is an important glycoprotein produced in the thyroid gland that acts as a precursor for the synthesis and storage of thyroid hormones, specifically, T4 and T3 [41,42]. The synthesis of these hormones includes the iodination of tyrosine residues within Tg, followed by proteolytic cleavage to release the active thyroid hormones into circulation [43,44]. Furthermore, thyroglobulin indirectly regulates glucose homeostasis; thyroid hormones generated from Tg have a key role in metabolic processes, such as gluconeogenesis and glucose uptake in diverse tissues, thereby altering overall glucose metabolism [31,45,46].

In the context of T2DM, the pathophysiology surrounding Tg concentration becomes more complex and the literature includes cases in which altered thyroid function leads to a decreased concentration of Tg [47]. This decrease may be attributed to several factors including the presence of insulin resistance, autoimmune damage, or chronic metabolic stress [48]. As autoimmunity increases (which can be indicated by TPOAb), thyroid cells may be progressively destroyed leading to reduced Tg synthesis and secretion [49]. Indeed, the results exhibited in the literature align with the findings of this study that presents a statistically significant decreased concentration of Tg in the T2DM group when compared to the non-prediabetic group.

The results further showed significantly reduced Tg concentrations in the prediabetic group in comparison with the non-prediabetic group. Interestingly, this trend was maintained when we compared the type 2 diabetic group with the prediabetic group. Evidence suggests that people in this transitional state may encounter metabolic disruptions where the reduced Tg concentration could also be indicative of thyroid hormone synthesis and storage capacity. For instance, insulin resistance, a hallmark of prediabetes, can cause changes in thyroid hormone metabolism, potentially leading to lower Tg concentrations as the body seeks to adjust for metabolic dysregulation. This compensatory strategy may show as an initial rise in TSH levels, which is also depicted in this study, followed by a decrease in Tg as the thyroid gland tries to maintain adequate hormone synthesis in the face of metabolic stress. The link between TSH and Tg in a prediabetic state suggests that increased TSH concentrations may correlate with changes in Tg, possibly reflecting thyroid dysfunction caused by metabolic abnormalities [50,51,52]. In this study, we observed an increase in TSH and a decrease in Tg in patients with prediabetes, and this observation was significantly prominent in patients with T2DM. This suggests a complicated interplay in which thyroid hormone regulation may change as insulin resistance progresses.

Thyroid hormones, specifically triiodothyronine (T3) and thyroxine (T4), play an important role in regulating metabolic processes in the body, such as the basal metabolic rate and energy expenditure [2,28]. T4 is primarily produced by the thyroid gland and is transformed into the more active T3 in peripheral tissues [53,54]. T3 exerts its effects by binding to thyroid hormone receptors, which influence gene expression and metabolic pathways [55]. In terms of glucose homeostasis, T3 improves glucose uptake by increasing the expression of glucose transporters and activating gluconeogenesis, both of which contribute to normal blood glucose levels. However, pathophysiological alterations might disrupt this balance [56,57].

Studies have shown that, as T2DM progresses, there is a significant reduction in total T3 and T4 concentrations [58,59,60]. Furthermore, insulin’s role as an anabolic hormone can influence thyroid hormone concentrations; it has been shown to enhance T4 concentration while suppressing T3 concentration, further complicating the hormonal interplay in patients with diabetes [60]. For instance, elevated concentrations of free fatty acids and inflammatory cytokines associated with insulin resistance can inhibit the activity of deiodinases, thereby reducing the conversion of T4 to T3 [61,62]. Indeed, the findings of this study demonstrate a modest drop in T3 and T4 concentration observed in the T2DM group compared to the non-prediabetic group, and this observation was additionally observed in the prediabetic group. It is interesting to note that a similar trend was observed in patients with prediabetes in this transitional state, as we observed non-significantly reduced concentration in patients with prediabetes when compared to the non-prediabetic group. Patients with prediabetes may experience metabolic disturbances that can disrupt the conversion of T4 to T3, resulting in a decreased T3 concentration while TSH concentration may begin to rise as a compensatory response.

FT3, the biologically active form of thyroid hormone, enhances gluconeogenesis in the liver, promoting the release of glucose into the bloodstream, while FT4 serves as a prohormone that is converted to FT3 in peripheral tissues and is involved in the regulation of glucose transporters and insulin sensitivity [63,64]. FT3 stimulates the transcription of genes involved in glycolysis and gluconeogenesis, thereby influencing blood glucose levels [65].

In the context of T2DM, FT3 levels tend to decrease while FT4 levels increase, reflecting the fact that higher FT4 levels correlate with insulin resistance, whereas lower FT3 levels are associated with impaired glucose metabolism [12,66]. However, there are also studies that report contradictory findings, indicating that some individuals may exhibit elevated FT3 levels in the context of insulin resistance which leans toward hyperthyroidism [67].

Certainly, the findings of this study exhibited significantly decreased concentrations of FT3 and significantly increased concentrations of FT4 in the T2DM group in comparison with the non-prediabetic control, and these results were also found in the prediabetic group.

During prediabetes, the conversion of FT4 to FT3 is impaired due to decreased activity of type 1 deiodinase (DIO1) and type 2 deiodinase (DIO2), and this can be attributed to the oxidative stress associated with insulin resistance [68,69].

The relationship between TSH, Tg, T3, T4, FT3, and FT4 in the prediabetic group is illustrated by a feedback process in which the body attempts to compensate for the altered metabolic status by increasing TSH levels in response to lower T3 and T4 levels [40,66]. An impairment in the conversion of T4 to T3 leads to a relative increase in FT4 levels as the thyroid gland compensates by producing more T4, while, simultaneously, the conversion to FT3 is reduced [70]. This can result in decreased thyroglobulin synthesis. Particularly, when insulin resistance progresses, it can influence TSH secretion and, in turn, the production of T3 and T4, which, in turn, affects thyroglobulin concentration and ultimately leads to thyroid dysfunction [71]. Similar findings were found in this study where patients with prediabetes had non-significantly higher TSH, T3, and T4 concentrations, which became considerably elevated with the switch to T2DM. Additionally, both prediabetic and T2DM groups had significantly higher concentrations of thyroglobulin. The activity of TPO is essential for the synthesis of thyroid hormones as iodine cannot be incorporated into Tg, preventing the formation of T3 and T4 [26]. Since TPO is more inclined to stimulate the coupling of DIT-to-DIT residues than the alternative DIT to MIT, studies have found that T4 is the more abundant thyroid hormone [26,72]. The autoimmune system’s lymphocytes produce TPO antibodies, which interfere with the action of TPO enzymes [26,73]. The dysregulation of immune function linked to T2DM is thought to be the cause of the elevated thyroid peroxidase (TPOAb) concentration in T2DM patients [74]. This heightened autoimmune response includes the creation of TPOAb [73,75]. Research has shown that individuals with T2DM have an elevated TPOAb concentration, reflecting an autoimmune response that targets thyroid tissue [75]. The autoimmune activity that has been observed in the literature and the present study in T2DM individuals can lead to thyroiditis, resulting in the destruction of thyroid follicular cells [31,76]. Indeed, the findings of this study present a significant increase in the TPOAb concentration measured in patients with T2DM in comparison with the non-prediabetic group. These results further showed that TPOAb concentrations were significantly increased in the prediabetic group when compared to the non-prediabetic group. This significant increase in TPOAb levels was also detected in the prediabetic group in comparison with the T2DM group. The findings of this study corroborated with the literature, indicating that the presence of an increased TPOAb concentration, even when it remains within the normal range, may signal a sub-clinical autoimmune response that could predispose individuals to thyroid dysfunction as they transition from the prediabetic phase to a type 2 diabetic state [15,31,77]. Additionally, a recent study conducted by Pillay et al. has shown that TPOAb concentrations are elevated in prediabetes because of immunological dysregulation that frequently follows metabolic disruptions, which intensifies autoimmune reactions against thyroid antigens [26]. In the prediabetic group, we observed the fact that the combination of elevated TSH and elevated TPOAb, along with decreased T3, T4, FT3, and Tg and increased FT4, raises the possibility of a compensatory condition that could result in subclinical thyroid dysfunction, including Hashimoto’s thyroiditis and subclinical hypothyroidism. Since these markers may point to an early stage of thyroid dysfunction that needs more research and treatment to stop the development of more severe thyroid illness, it is imperative to monitor them in patients with prediabetes.

## 4. Methods and Materials

### 4.1. Study Design and Setting

This study followed a retrospective study design and was carried out in a Durban-based clinical setting of eThekwini Metropolitan, South Africa. Ethical approval was obtained from the University of KwaZulu Natal (UKZN) Biomedical Research Ethics Committee (BREC) (Study ref: BE266/19) as well as the Provincial Health Research Committee from the KZN Department of Health. This study was carried out in collaboration with the King Edward Hospital (KEH), a tertiary-level hospital that provides services to the residents of the KZN province. Samples were only collected from patients once informed consent was obtained.

### 4.2. Prediabetes Diagnosis and Eligibility Criteria

To confirm the glycaemic status of each sample, samples were categorized as non-diabetic, prediabetic, and type 2 diabetic based on the American Diabetes Association (ADA) diabetes diagnostic criteria. Prediabetes was defined as HbA1c values ranging from 5.7 to 6.4% and impaired fasting glucose (IFG) concentrations between 5.6 and 6.9 mmol/L, according to the ADA criterion.

The inclusion criteria comprised samples collected from patients of both genders aged between 25 and 45 years of all ethnicities. The exclusion criteria excluded those who had no thyroid surgery or trauma to the neck. Patients diagnosed with thyroid disorders or on anti-thyroid drugs or previously diagnosed with gestational diabetes and type 1 diabetes mellitus were excluded from this study. Additionally, patients with a history of liver and kidney disease, those who have severe infectious disease as well as pregnant women were not eligible for this study.

### 4.3. Sample Processing

Blood samples were collected in EDTA tubes from participants who confirmed overnight fasting and met the eligibility criteria. The fasting plasma glucose was measured using a glucometer. The glycated hemoglobin (HbA1c) was measured using a clinically validated NGSP and IFCC-certified, laboratory-based Tosoh G8 HPLC Analyzer. This study utilized a total of 80 blood samples from various patients based on HbA1c parameters. The samples were categorized into three groups: non-diabetic (ND, *n* = 26), prediabetic (PD, *n* = 27), and type 2 diabetes mellitus (T2DM, *n* = 27).

The samples were then centrifuged (Eppendorf Centrifuge 5405, Germany) to collect serum and plasma and then stored in an 80 °C Bio freezer (Snijders, Scientific, Holland) for further biochemical analysis.

This study utilized a total of 80 blood samples from various patients. Based on HbA1c parameters, the samples were categorized into three groups: non-diabetic (ND, *n* = 26), prediabetic (PD, *n* = 27), and type 2 diabetes mellitus (T2DM, *n* = 27).

### 4.4. Biochemical Analysis

The thyroid stimulating hormone (TSH), thyroxine (T4), triiodothyronine (T3), thyroglobulin (TG), and thyroid peroxidase antibody (TPOAb) concentrations were determined by using their respective ELISA kits (Abclonal, Woburn, MA, USA) using the manufacturer’s instructions. The optical densities were measured spectrophotometrically at wavelengths of 450 nm and 570 nm using a nano spectrophotometer (BMG Labtech, Passau, Germany). The concentrations of the plasma TSH, T3, T4, TG, and TPOAb within the samples were extrapolated from their respective standard curves.

### 4.5. Statistical Analysis

All data are expressed as means ± standard deviation (SD). A normality test was conducted, after which data were analyzed using the GraphPad Prism InStat Software (version 8.00, GraphPad Software, San Diego, CA, USA). The terminal parameters were analyzed using a one-way analysis of variance (ANOVA) followed by a Tukey Kramer post-hoc test to determine the statistical differences between the control and experimental groups. Values of *p* < 0.05 were considered statistically significant.

## 5. Conclusions

Taken together, the findings from this population suggest that thyroid dysfunction is an important contributor to the progression of prediabetes. During the prediabetic phase, Tg concentration and TPOAb levels change significantly, indicating underlying thyroid stress. Prediabetes has been found to be a compensatory state for many endocrinological functions, and, in this study, we observed a TSH increase in an attempt to stabilize thyroid hormone levels. However, as patients transition to T2DM, further imbalances in these hormones become more pronounced.

## 6. Limitations and Future Studies

The retrospective methodology of this study is one of its limitations, which might have produced evidence that was not entirely clear about the degree of thyroid dysfunction. Subsequently, this opens an area of research for future prospective observational studies to longitudinally assess whether thyroid dysfunction precedes or influences the development of prediabetes.

Furthermore, due to the retrospective nature of this study, key founding variables, such as oral glucose tolerance test (OGTT), body mass index (BMI), iodine intake, vitamin D status, and dietary and nutritional intake, were not adequately accounted for. These variables also play a significant role in thyroid function, and their absence from analysis may limit the depth of this study’s overall conclusion. A future prospective observational study can aim for key confounding variables such as BMI, iodine intake, and dietary intake to provide a more accurate assessment of the relationship between thyroid dysfunction and prediabetes. Subsequent studies can further address the limitation of sample size and gender disparity by recruiting a larger, and more diverse cohort to improve the statistical power and increase the ability to identify significant associations between thyroid dysfunction and prediabetes.

## Data Availability

Data are contained within the article.

## Abbreviations

TSH	Thyroid Stimulating Hormone
T4	Thyroxine
T3	Triiodothyronine
TG	Thyroglobulin
TPOAb	Thyroid Peroxidase Antibody
TPO	Thyroid Peroxidase
UKZN	University of KwaZulu Natal
BREC	Biomedical Research Ethics Committee
ADA	American Diabetes Association
NPD	Non-Prediabetic
PD	Prediabetic
T2DM	Type 2 Diabetes Melittus
HbA1c	Glycated Haemoglobin
IFG	Impaired Fasting Glucose
OGTT	Oral Glucose Tolerance Test
BMI	Body Mass Index
IGT	Impaired Glucose Tolerance
SD	Standard Deviation
IQR	Interquartile Range
CI	Confidence Interval

## Appendix A

**Table A1:** Thyroid hormone changes stratified according to gender.

Hormone (Units)	NPD	PD	T2DM
Thyroid Stimulating Hormone (mIU/L)			
**Male**	4.362 (3.801–4.924)	4.592 (3.204–5.980)	5.253 (4.348–6.159)
**Female**	4.23 (3.75–4.708)	4.700 (4.323–5.077)	4.916 (4.320–5.513)
Triiodothyronine (nmol/L)			
**Male**	1.483 (1.358–1.608)	1.551 (1.350–1.752)	1.552 (1.442–1.662)
**Female**	1.579 (1.443–1.1715)	1.379 (1.249–1.509)	1.450 (1.334–1.567)
Thyroxine (ug/dL)			
**Male**	9.417 (7.882–10.95)	8.299 (6.982–9.615)	8.098 (6.840–9.356)
**Female**	8.792 (8.072–9.513)	8.914 (8.367–9.461)	7.861 (6.615–9.107)
Thyroglobulin (ng/mL)			
**Male**	21.84 (16.09–27.59)	13.86 (7.733–19.99)	7.907 (4.563–11.25)
**Female**	23.75 (21.21–26.29)	13.49 (10.78–16.20)	7.548 (5.839–9.257)
Thyroid Peroxidase Antibody (IU/mL)			
**Male**	17.02 (14.46–19.57)	18.73 (14.03–23.42)	22.97 (20.11–25.83)
**Female**	18.60 (16.22–20.98)	19.53 (16.96–22.11)	23.16 (20.99–25.33)

The data are presented as mean (95% CI) or median (IQR) depending on the normality and proportion.
